# On the relevance of technical variation due to building pools in microarray experiments

**DOI:** 10.1186/s12864-015-2055-6

**Published:** 2015-12-01

**Authors:** Henrik Rudolf, Gerd Nuernberg, Dirk Koczan, Jens Vanselow, Tanja Gempe, Martin Beye, Gérard Leboulle, Kaspar Bienefeld, Norbert Reinsch

**Affiliations:** Institut für Genetik und Biometrie, Leibniz-Institut für Nutztierbiologie, Dummerstorf, DE Germany; Institut für Immunologie, Universität Rostock, Rostock, DE Germany; Institut für Evolutionsgenetik, Heinrich Heine Universität Düsseldorf, Düsseldorf, DE Germany; Neurobiologie, Freie Universität Berlin, Berlin, DE Germany; Länderinstitut für Bienenkunde Hohen Neuendorf, Hohen Neuendorf, DE Germany

## Abstract

**Background:**

Pooled samples are frequently used in experiments measuring gene expression. In this method, RNA from different individuals sharing the same experimental conditions and explanatory variables is blended and their concentrations are jointly measured. As a matter of principle, individuals are represented in equal shares in each pool. However, some degree of disproportionality may arise from the limits of technical precision. As a consequence a special kind of technical error occurs, which can be modelled by a respective variance component. Previously published theory - allowing for variable pool sizes - has been applied to four microarray gene expression data sets from different species in order to assess the practical relevance of this type of technical error in terms of significance and size of this variance component.

**Results:**

The number of transcripts with a significant variance component due to imperfect blending was found to be 4329 (23 %) in mouse data and 7093 (49 %) in honey bees, but only 6 in rats and none whatsoever in human data. These results correspond to a false discovery rate of 5 % in each data set. The number of transcripts found to be differentially expressed between treatments was always higher when the blending error variance was neglected. Simulations clearly indicated overly-optimistic (anti-conservative) test results in terms of false discovery rates whenever this source of variability was not represented in the model.

**Conclusions:**

Imperfect equality of shares when blending RNA from different individuals into joint pools of variable size is a source of technical variation with relevance for experimental design, practice at the laboratory bench and data analysis. Its potentially adverse effects, incorrect identification of differentially expressed transcripts and overly-optimistic significance tests, can be fully avoided, however, by the sound application of recently established theory and models for data analysis.

**Electronic supplementary material:**

The online version of this article (doi:10.1186/s12864-015-2055-6) contains supplementary material, which is available to authorized users.

## Background

In gene expression profiling pooling is a method to reduce hybridization costs and compensate for insufficient amounts of mRNA. In the subsequent statistical analyses of gene expression data, where a log-transformation during preprocessing is standard, it is important to consider how the expectation and variance of the gene expression of pools relate to individual samples. The impact of pooling on the identification of differential gene expression has been studied in Kendziorski et al. [1], separately for different pool sizes. It has been shown that biological averaging occurs for most of the transcripts and differential expression inferences are comparable for individuals and pools. In Zhang et al. [2] approximations for the expectation and variance of pooled samples were derived. Furthermore, it was shown that biases as well as heteroscedasticity are introduced by variable pool sizes. Experiments with unequal pool sizes therefore were recommended to be avoided. As demonstrated in Rudolf et al. [3], however, a wide class of experiments, in which pool size can be handled as a nuisance effect and is cross-classified with treatment, allows for tests of unbiased contrasts. In the case of a balanced cross-classification the pool size effect must not explicitly appear in the model at all, though hypotheses on treatments remain unbiased, as shown in Rudolf et al. [3]. In any case variable pool sizes have an effect on the covariance of observations. This can be taken into account by considering how many individuals are allocated to each pool and by introducing a random effect for blending along with a corresponding variance component. The latter can be interpreted as a second kind of technical variability induced by inaccuracies in blending slightly unequally-sized aliquots of mRNA from several individuals into common pools. Though this subject has been treated theoretically as described, investigation into the practical importance of this second kind of technical variability is lacking.

Consequently a study was performed, in which gene expression data from experiments with four different species were analyzed to investigate the relevance of the aforementioned new kind of technical error in terms of size and significance of the corresponding variance component. Furthermore, we investigated potential consequences on the number of transcripts identified as differentially expressed between different treatments when analyses neglect this kind of error.

## Methods

This section offers a short recap of the underlying statistical models. The four experimental data sets are then introduced. In all of them - whether from single- or two-color arrays - there are more observations than pools (see Table 1), which allows for the estimation of all desired variance components. Data simulations are also described and have been included as a useful aid for the interpretation of the experimental data results. Finally, the statistical methods applied for parameter estimation and statistical testing are described.

**Table 1:** Characteristics of experimental data sets

Characteristics	Mouse	Rat	Bee	Human
Individuals	60	24	14	55
Pools	12	22	12	16
Pool size	5	2,3,12	2,4	3
Observations	44	56	22	30
1-/2- color-array	1	1	2	2

### Random effects in gene expression experiments with variable pool sizes

When aliquots of mRNA from different individuals are blended into common pools, the inaccuracies of this procedure may induce a special kind of technical error. Respective random effects, together with a corresponding variance component, were proposed [3] as a means of modeling the variability of pooled observations in gene expression experiments with variable pool sizes (i.e. differing numbers of individuals per pool). Thus, for background-corrected and normalized log-intensities **y** (length of vector **y** equals the number of arrays) of a certain transcript, the model in matrix notation is:

(1) $$ \mathbf{y}=\mathbf{X}\boldsymbol{\upbeta}+\mathbf{Z}_{1}\mathbf{u}_{1}+\mathbf{Z}_{2}\mathbf{u}_{2}+\mathbf{e},  $$

where **X** and **Z** are the design matrices of the fixed (***β***=(*μ*,*β*_*t*_)^⊤^) and random (**u**_1_,**u**_2_) effects. The distribution of **u**_*j*_ is assumed to be $\mathbf {u}_{j}\sim N(\mathbf {0},\mathbf {G}_{j}{\sigma _{j}^{2}}),\ j=1,2$ with covariance matrices $\mathbf {G}_{j}{\sigma _{j}^{2}}$ (${\sigma _{j}^{2}}$ are the variance components) and the residuals are $\mathbf {e}\sim N(0,\mathbf {I}{\sigma _{e}^{2}})$. Random effects of single individuals are assumed to be independently identically distributed with a biological variance ${\sigma _{1}^{2}}$, while observations from a number of *γ*_*i*_ pooled individuals have a biological variance $\frac {{\sigma _{1}^{2}}}{\gamma _{i}}$. The vector *u*_1_ may comprise biological effects of single individuals as well as average biological effects of groups of individuals constituting common pools, according to the experimental design.

The random effect of blending (i.e. for the technical procedure of building a pool) only applies to observations from pools and not to observations from single individuals. Therefore, *u*_2_ consists of one effect per mixture, which had been prepared in the lab. The associated variance component is ${\sigma _{2}^{2}}$. So, the variance of the observations becomes:

(2) $$ \mathbf{V}(\mathbf{y})=\mathbf{Z}_{1}^{}\mathbf{G}_{1}^{}\mathbf{Z}_{1}^{\top}{\sigma_{1}^{2}}+\mathbf{Z}_{2}^{}\mathbf{G}_{2}^{}\mathbf{Z}_{2}^{\top}{\sigma_{2}^{2}}+\mathbf{I}_{n}{\sigma_{e}^{2}}.  $$

The model of this variance structure is based on the closed form approximation of the variance of pools on the scale of log-intensities, proposed in [2]

(3) $${} v_{i}\approx\left(e^{{\sigma_{b}^{2}}}-1 \right)\frac{1}{\gamma_{i}}+\left(e^{{\sigma_{b}^{2}}}-1 \right){\sigma_{z}^{2}}\frac{\gamma_{i}-1}{{\gamma_{i}^{2}}},  $$

where ${\sigma _{z}^{2}}$ is the pooling technical variance and ${\sigma _{b}^{2}}$ is the biological variance of individuals. The substitutions ${\sigma _{1}^{2}}:=e^{{\sigma _{b}^{2}}}-1$ and ${\sigma _{2}^{2}}:=(e^{{\sigma _{b}^{2}}}-1){\sigma _{z}^{2}}$ led to our assumed variance structure (2).

In the following, the relevance of accounting for the blending error variance component ${\sigma _{2}^{2}}$ is investigated in four experimental data sets by comparing the described full model (m2) described above with a reduced one (m1) that lacks this particular variance component. The methodology was checked by a simulation beforehand.

### Experimental data

#### Mouse data

Mouse data consisted of observations from 44 one-color microarrays. RNA for this experiment was extracted from the ovaries of 60 female mice, 30 of which came from a long-term selection line with an extraordinary litter size. All others came from a control line. Pooled samples were built by blending RNA from five mice per sample. Each mouse was only represented in a single pool. For the sake of technical replication, all 12 pooled samples were measured twice by preparing two microarrays per sample. Additionally, animals from two pools per line (ten animals per line) were measured individually. These individual measurements were not included in the previously published analysis of this data [4], where more details of the experiment can be found.

Twenty-eight (14 per line) different biological effects were defined per transcript. The dimensions of the design matrix $\mathbf {Z_{1}^{m}}$ are therefore 44 × 28. In detail, random biological effects were assigned to all individually measured mice (individuals 1 to 10 within each line) and corresponding entries in $\mathbf {Z}_{1}^{m}$ equal 1. The biological effects of the same ten individuals (in two groups of five) were assigned to the observations from the first and second pooled samples in each line (two observations per pool due to technical replication). In this case, non-zero entries in $\mathbf {Z}_{1}^{m}$ are 1/5. However, pooled samples numbered three to six within each line each had a biological effect of their own, modeling the average effect of the five respective members of each pool. Note that for the pools three to six the corresponding non-zero entries in $\mathbf {Z}_{1}^{m}$ are 1. The 28×28 covariance matrix $\mathbf {G}_{1}^{m}$ scales the random biological effects and has non-zero entries only on the diagonal, each of them equals the inverse pool size 1/*γ*_*i*_. The 22 observations from the first line are represented in the upper part of the design matrix $\mathbf {Z}_{1}^{m}$:

$$\begin{array}{@{}rcl@{}} \mathbf{Z}_{1}^{m}= \left[\begin{array}{cccccccccccccccccccccccccccc} 1&0&0&0&0&0&0&0&0&0&0&0&0&0&0&0&0&0&0&0&0&0&0&0&0&0&0&0\\ 0&1&0&0&0&0&0&0&0&0&0&0&0&0&0&0&0&0&0&0&0&0&0&0&0&0&0&0\\ 0&0&1&0&0&0&0&0&0&0&0&0&0&0&0&0&0&0&0&0&0&0&0&0&0&0&0&0\\ 0&0&0&1&0&0&0&0&0&0&0&0&0&0&0&0&0&0&0&0&0&0&0&0&0&0&0&0\\ 0&0&0&0&1&0&0&0&0&0&0&0&0&0&0&0&0&0&0&0&0&0&0&0&0&0&0&0\\ 0&0&0&0&0&1&0&0&0&0&0&0&0&0&0&0&0&0&0&0&0&0&0&0&0&0&0&0\\ 0&0&0&0&0&0&1&0&0&0&0&0&0&0&0&0&0&0&0&0&0&0&0&0&0&0&0&0\\ 0&0&0&0&0&0&0&1&0&0&0&0&0&0&0&0&0&0&0&0&0&0&0&0&0&0&0&0\\ 0&0&0&0&0&0&0&0&1&0&0&0&0&0&0&0&0&0&0&0&0&0&0&0&0&0&0&0\\ 0&0&0&0&0&0&0&0&0&1&0&0&0&0&0&0&0&0&0&0&0&0&0&0&0&0&0&0\\ 0.2&0.2&0.2&0.2&0.2&0&0&0&0&0&0&0&0&0&0&0&0&0&0&0&0&0&0&0&0&0&0&0\\ 0.2&0.2&0.2&0.2&0.2&0&0&0&0&0&0&0&0&0&0&0&0&0&0&0&0&0&0&0&0&0&0&0\\ 0&0&0&0&0&0.2&0.2&0.2&0.2&0.2&0&0&0&0&0&0&0&0&0&0&0&0&0&0&0&0&0&0\\ 0&0&0&0&0&0.2&0.2&0.2&0.2&0.2&0&0&0&0&0&0&0&0&0&0&0&0&0&0&0&0&0&0\\ 0&0&0&0&0&0&0&0&0&0&1&0&0&0&0&0&0&0&0&0&0&0&0&0&0&0&0&0\\ 0&0&0&0&0&0&0&0&0&0&1&0&0&0&0&0&0&0&0&0&0&0&0&0&0&0&0&0\\ 0&0&0&0&0&0&0&0&0&0&0&1&0&0&0&0&0&0&0&0&0&0&0&0&0&0&0&0\\ 0&0&0&0&0&0&0&0&0&0&0&1&0&0&0&0&0&0&0&0&0&0&0&0&0&0&0&0\\ 0&0&0&0&0&0&0&0&0&0&0&0&1&0&0&0&0&0&0&0&0&0&0&0&0&0&0&0\\ 0&0&0&0&0&0&0&0&0&0&0&0&1&0&0&0&0&0&0&0&0&0&0&0&0&0&0&0\\ 0&0&0&0&0&0&0&0&0&0&0&0&0&1&0&0&0&0&0&0&0&0&0&0&0&0&0&0\\ 0&0&0&0&0&0&0&0&0&0&0&0&0&1&0&0&0&0&0&0&0&0&0&0&0&0&0&0 \\&&&&&\vdots&&&&&&&\vdots&&&&&&&\vdots&&&&&&&\vdots& \end{array}\right]_. \end{array} $$

The technical variability due to blending individual samples only comes into play when observing pooled samples, not for measurements of individuals. Since blending was done only once per pool, there are 12 different effects due to imperfect blending. Therefore, the 44×12 design matrix $\mathbf {Z}_{2}^{m}$ (see Additional file 1) contains zero rows for observations from single animals. The corresponding 12×12 covariance matrix $\mathbf {G}_{2}^{m}$ is diagonal with entries $\frac {\gamma -1}{\gamma ^{2}}=\frac {4}{25}$, according to Eq. (3).

This study did not involve in vivo experiments. Animals were housed according to the German law for animal protection (TierSchG) and in compliance with the European legislation on the care and use of animals.

#### Rat data

This data set was analyzed by Kendziorki et al. [1] and contains one-color array data. Rats of the treatment group were treated with Retinoic acid. For the details of data generation and preprocessing, please see the original paper [1]. Rats from the groups A (control) and B (treatment) were measured individually and in pools of various sizes. Each of the twelve rats from both groups was used four times, for an individual measurement and in pools of 2, 3, and 12. For the sample composition we again defined the random effects from the smallest disjunct elements. Therefore, with the help of the matrices $\mathbf {G}_{1}^{r}$ and $\mathbf {Z}_{1}^{r}$, convex linear combinations were built from the 24 individuals. Here, $\mathbf {G}_{1}^{r}$ is the 24 × 24 unity matrix and $\mathbf {Z}_{1}^{r}$ contains a row for each measurement with entries according to reciprocal pool sizes. Per group, there are 28 measurements partitioned into 12 individual samples, 6 pools of 2, 4 pools of 3, and one of 12, plus 5 technical replications. Thus, the dimensions of the matrix $\mathbf {Z}_{1}^{r}$ are 56×24, detailed in the Additional file 1. In each group, there were 11 pools, and the diagonal matrix $\mathbf {G}_{2}^{r}$ has the dimensions 22×22 with entries $\left \{\frac {2}{9},\frac {1}{4},\frac {2}{9},\frac {1}{4},\frac {2}{9},\frac {1}{4},\frac {1}{4},\frac {1}{4},\frac {2}{9},\frac {1}{4},\frac {11}{144},...\right \}$. The matrix $\mathbf {Z}_{2}^{r}$ was constructed analogously to $\mathbf {Z}_{1}^{r}$.

#### Honey bee data

**Fig. 1:**
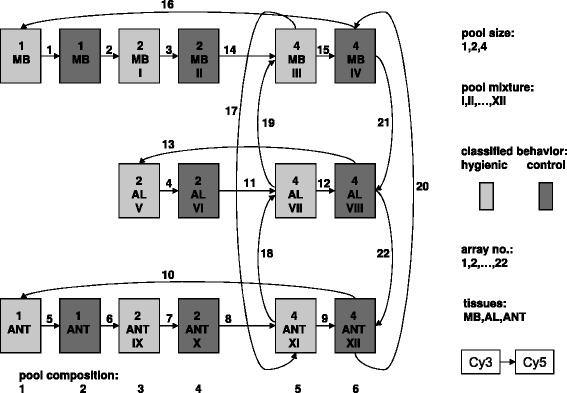
Design of the honey bee experiment. Scheme for the design of the two-color microarray experiment with honey bees. The numbered arrows (1–22) represent two-color arrays, the arrowheads (tails) indicate Cy5 (Cy3) dye. Light (dark) boxes symbolize RNA from hygienic (control) bees. Pool size (1, 2, 4) and mixture (Roman numerals) are shown in each box. Tissues are abbreviated as MB (mushroom body), AL (antennal lobe), and ANT (Antennae). Boxes in the same column share the same biological effect, indicated as pool compositions 1 to 6

This data set stems from a honeybee project dealing with differences in the pathogen resistance of so-called hygienic and non-hygienic worker bees as far as they are reflected in gene expression differences. Bees designated as ’hygienic’ were observed to open brood cells and assisting the removal of diseased brood. The bees’ activities were recorded on a *Varroa*-parasitized section of a brood comb. Pooling was applied in a preliminary experiment with a limited number of bees and microarrays. For seven hygienic bees and seven control bees, mRNA was extracted from nerve tissues of the mushroom body (MB), antennal lobe (AL) and Antennae (ANT). The number of individuals blended into a pool was either two or four. Out of the 14 bees, six different sample compositions were built and analyzed for all three tissues with two-color arrays (for the design see Fig. 1). A few individual hybridizations were not carried out due to an insufficient amount of amplified RNA (single samples from AL). For the normalized two-color microarray data we used a model for differences **M** of log-intensities from the red (R) and green (G) channel

(4) $$ \mathbf{M}=\mu+\boldsymbol\Delta+b_{12}+b_{23}+\mathbf{Z}_{1}^{}\mathbf{u}_{1}^{}+\mathbf{Z}_{2}^{}\mathbf{u}_{2}^{}+\mathbf{e}.  $$

Here **M** is the vector of log-ratios ($\mathbf {M}=\log \frac {R}{G}=\log R-\log G$) for one transcript with dimension *n*, equal to the number of arrays. The design matrix **X** for the fixed effects links observations to the overall mean *μ* (which includes the dye effect, i.e. the difference of red and green channel), the differences ***Δ*** between the behaviors (hygienic minus control) and two differences between tissues (*b*_12_ for MB minus AL, *b*_23_ for AL minus ANT). The latter effect has been included since data from all tissues were jointly analyzed due to the limited number of arrays. The random effect **u**_1_ for each sample composition has a variance structure determined by $\mathbf {G}_{1}^{h}$ and $\mathbf {Z}_{1}^{h}$. The variance structure of the second random effect **u**_2_ for the blending of individuals is generated by $\mathbf {G}_{2}^{h}$ and $\mathbf {Z}_{2}^{h}$. Both design matrices for the random effects differ, however, from experiments with one-color arrays: each row of **Z**_1_ and **Z**_2_ contains two non-zero elements (as opposed to a single one) in order to model the differences between effects with entries of 1 for the red and −1 for the green channel. The residual errors $\mathbf {e}\sim N(0,{\sigma _{e}^{2}})$ are again assumed to be stochastically independent and include the technical errors created through the hybridization, imaging, and scanning of each array.

#### Human data

The human data was taken from the GC6 (Grand Challenge in Global Health no. 6 - Biomarkers of protective immunity against Tuberculosis) project. For the project data, please see Maertzdorf et al. [5] and Jacobsen et al. [6]. One focus of this project was to identify immune system differences between people who were exposed to Tuberculosis but never became sick and those who developed severe symptoms. Therefore, as a part of this larger study the three classes TST^+^, TST^−^ and TB were compared, where TST stands for the tuberculosis skin test (+ and - indicate positive and negative results, respectively) and TB for acute tuberculosis. Overall, the data set consists of samples from 55 humans in 16 pools of three and in ten single samples, which were labeled on 30 two-color arrays. In the sample composition, one also sees correlations between pools in three cases, where individuals were used more than once, i.e. in different pools (see matrix $\mathbf {G}_{1}^{g}$). For each observation we modeled fixed effects for the mean (including dye effect) and treatment (3 levels) as well as random effects of sample composition and imperfect blending. Because there were two samples on each array, the design matrix $\mathbf {Z}_{1}^{g}$ for the composition of the samples had two entries per row, as presented in the Additional files 1. Each pool was built only once, so $\mathbf {G}_{2}^{g}$ is a diagonal matrix with dimensions 16×16 and entries $\frac {2}{9}$. The random effects of imperfect blending were assigned to measurements via $\mathbf {Z}_{2}^{g}$, with two non-zero entries per measurement.

This study was approved by ethical committees in both Stellenbosch (South Africa) and Berlin (Germany) and written informed consent has been obtained from all study participants (details in [6]).

### Simulated data

The relationship between the variance of a random effect of a pool and deviations from the homogene aliquots of individuals in a pool sample, given in Eq. (3), is based on a theoretically derived approximation [2]. Furthermore, true proportions of aliquots are not available. Therefore, the equality of the estimated variance component ${\sigma _{2}^{2}}$ and the product of variances $(e^{{\sigma _{b}^{2}}}-1){\sigma _{z}^{2}}$ was checked by fitting the model to simulated data, in order to assay the estimations when the true state of nature is known.

By setting $\mathbf {x}\sim N(\mu _{g},\mathbf {I}{\sigma _{b}^{2}})$ the vector of individual gene expressions of the individuals of a pool and **w** the vector of weights (proportions of individuals in the pooled RNA of a joint sample), we calculated a value for true gene expression on the log-scale as

(5) $$ \log\left(\mathbf{w}^{\top}\times\exp\left(\mathbf{x}\right)\right).  $$

The technical errors, distributed as $N(0,{\sigma _{t}^{2}})$, were then added. Note that, due to (1), each observation is composed by the fixed effects **X*β***=*μ*_*g*_, the distortion due to biological variation $\mathbf {u}_{1}=\bar {\mathbf {x}}-\mu _{g}$ and the difference generated by imperfect blending $\mathbf {u}_{2}=\log (\mathbf {w}^{\top }\times \exp \left (\mathbf {x})\right)-\log \left (\overline {\exp \left (\mathbf {x}\right)}\right)$, plus the log-bias $\log \left (\overline {\exp \left (\mathbf {x}\right)}\right)-\bar {\mathbf {x}}$. For the simulation of weights the Dirichlet distribution with parameters $a_{i}=\frac {1}{{\sigma _{z}^{2}}}-\frac {1}{\gamma }$, *i*=1,…,*γ* was used. Then, $a_{0}=\sum \limits _{i=1}^{\gamma }{a_{i}}=\gamma a_{i}$, and the expectation of each weight is $\frac {a_{i}}{a_{0}}=\frac {1}{\gamma }$. Therefore, the variance of the weights - theoretically $\frac {a_{i}(a_{0}-a_{i})}{{a_{0}^{2}}(a_{0}+1)}$ - is $\frac {\gamma -1}{\gamma ^{3}}{\sigma _{z}^{2}}$. Using the approximation $\frac {\gamma -1}{\gamma ^{3}}{\sigma _{z}^{2}}\approx {\sigma _{w}^{2}}$ for the variance of weights **w** from [2], the Dirichlet parameters *a*_*i*_ can be chosen in order to obtain weights with a given variance ${\sigma _{w}^{2}}$.

Various proportions of transcripts (0, 1/3, 1) were simulated as affected by imperfect blending. In order to investigate the distribution of the RLRT-statistic under the null hypothesis (${\sigma _{2}^{2}}=0$), the pooling technical variance ${\sigma _{z}^{2}}$ was set to zero for all transcripts. Then, one third of the transcripts were simulated with imperfect blending, as well as data where all transcripts contained these effects.

As a test case, further simulations were tailored for a comparison of models with regard to the power to detect differential expression in the presence of imperfect pooling at all loci. Variances were set to ${\sigma _{t}^{2}}=0.17$, ${\sigma _{b}^{2}}=0.103$ and ${\sigma _{z}^{2}}=2.7$ according to the estimations from the mouse data. This was simulated with 100 repetitions. An experiment consisting of 60 individuals from two equally-sized treatment groups was simulated, in a 44 one-color microarray setting. The observations generated were both from single individuals (20) and pools of size five (24). The individual values used in the first two pools of each line were also used as single individuals. For the full details of the design, please see the description of the mouse data set above, which has an identical structure. For each of the 9000 transcripts, a mean expression level was randomly chosen from a uniform distribution over the interval [8,14]. A subgroup of 3000 transcripts was randomly chosen to be differentially expressed between both treatment groups. For each of these, a mean treatment effect was sampled from a uniform distribution over the interval [0.5,1.5] with a random sign ∈{−1,1}. False positive and negative test results were then evaluated using the mean number of transcripts, averaged over all 100 repetitions.

### Statistical analyses

Three variance components were considered: first, biological variance (${\sigma _{1}^{2}}$); second, blending error variance (${\sigma _{2}^{2}}$); and third, residual variance (${\sigma _{e}^{2}}$). Similar models that lack the second variance component have been used previously (e.g. [7]). Transcripts were excluded from analyses if the log-expressions of both groups were smaller than eight (corresponds to 256 at the original scale), which is frequently considered to be a threshold for meaningful gene expression. This resulted in 8554 observations for the mouse data, 6264 for rats, 13,761 for bees and 12,348 for the human data set. An EM-REML algorithm was used to estimate the variance components. Then the mixed model equations

$${\small{\begin{aligned} {}\left[ \begin{array}{ccc} \mathbf{X}^{\top} \mathbf{X}&\mathbf{X}^{\top} \mathbf{Z}_{1}^{}&\mathbf{X}^{\top} \mathbf{Z}_{2}^{}\\ \mathbf{Z}_{1}^{\top} \mathbf{X}&\mathbf{Z}_{1}^{\top} \mathbf{Z}_{1}^{}+\mathbf{G}_{1}^{-1}\lambda_{1}^{}&\mathbf{Z}_{1}^{\top} \mathbf{Z}_{2}^{}\\ \mathbf{Z}_{2}^{\top} \mathbf{X}&\mathbf{Z}_{2}^{\top} \mathbf{Z}_{1}^{}&\mathbf{Z}_{2}^{\top} \mathbf{Z}_{2}^{}+\mathbf{G}_{2}^{-1}\lambda_{2}^{} \end{array}\right]\! \left[ \begin{array}{c} \hat{\boldsymbol\upbeta}\\ \hat{\mathbf{u}}_{1}\\ \hat{\mathbf{u}}_{2} \end{array}\right] \,=\, \left[ \begin{array}{c} \mathbf{X}^{\top} \mathbf{Y}\\ \mathbf{Z}_{1}^{\top} \mathbf{Y}\\ \mathbf{Z}_{2}^{2\top} \mathbf{Y} \end{array}\right]_, \end{aligned}}} $$

where $\lambda _{1}^{}=\frac {{\sigma _{e}^{2}}}{{\sigma _{1}^{2}}}$ and $\lambda _{2}^{}=\frac {{\sigma _{e}^{2}}}{{\sigma _{2}^{2}}}$, were solved for the estimates of the fixed and random effects and the REML-log-likelihood was calculated.

**Fig. 2:**
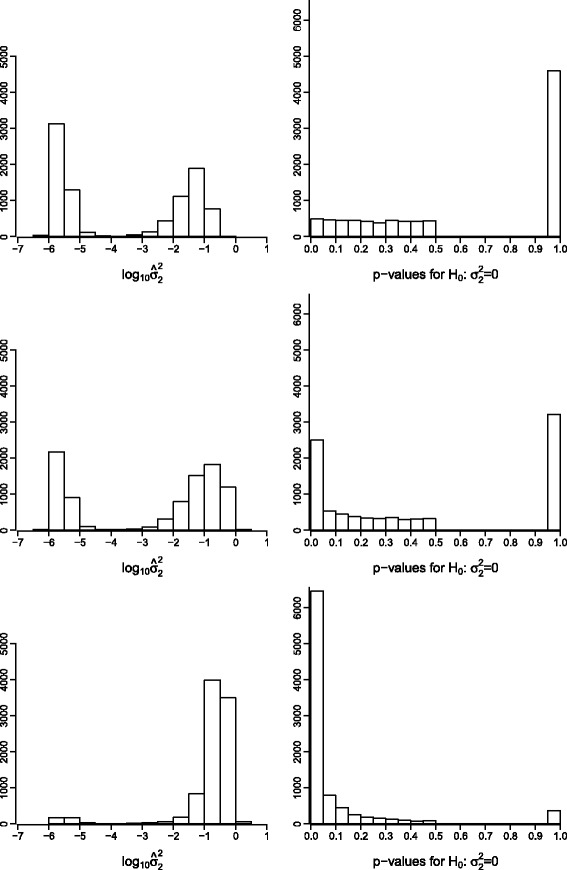
Estimates of blending error variance for simulated data. Log-estimates of the blending error variance ${\sigma _{2}^{2}}$ *(left)* and *p*-values *(right)* of RLRT ($\mathbf {H}_{0}:{\sigma _{2}^{2}}=0$) for simulated data. Top: perfectly blended individuals were simulated. The *p*-values of the interval [0,0.5) are uniformly distributed and nearly half of the transcripts have a *p*-value of 1. Middle: 3000 out of 9000 transcripts affected by imperfect blending of individuals. Bottom: all transcripts were simulated with imperfect blending

For each transcript, a residual likelihood ratio test (RLRT) was used to test the null hypothesis $H_{0}:{\sigma _{2}^{2}}=0$, thereby assuming a half-half mixture of a ${\chi _{1}^{2}}$-distribution and a point mass at zero (see e.g. [8]). According to this assumed distribution of the test statistic, the distribution of *p*-values from all transcripts in one experiment under the null hypothesis deviates from the uniform distribution (see Fig. 2). The proportion of transcripts with a relevant blending error variance was estimated as $\hat {\pi }_{1}=1-\hat {\pi }_{0}$. Therein, the estimated proportion of true null hypotheses ($\hat {\pi }_{0}$) was estimated as described in Dabney and Storey [9]. The proportion $\hat {\pi }_{1}$ was then compared with the proportion of transcripts simulated without blending errors. After correcting all *p*-values according to a false discovery rate (FDR) of 5 *%*, the transcripts with a significant RLRT were determined. Beyond that, we evaluated the proportions of the estimated variance component ${\sigma _{2}^{2}}$ in relation to the total variance.

The practical relevance of the variance component for imperfect blending of samples was further investigated by comparing the number of transcripts identified as differentially expressed in different treatment levels by means of the full model (m2, Eq. 1) and the null model (m1) **y**=**X*β***+**Z**_1_**u**_1_+**e** without a random effect of imperfect blending. Degrees of freedom for the applied F-Tests of fixed effects in mixed models were adjusted according to Kenward and Roger [10]. In order to account for multiple testing, an FDR of 5 *%* was applied to the *p*-values of the latter F-tests.

For the mouse data set, the normalization was done with the gcrma method [11]. Loess- and quantile normalization [12] was used for the two-color array data. The rat data set was downloaded as normalized.

The open-source statistical programming package R [13] was used to implement an EM-REML algorithm for the estimation of all three variance components. The formulas for the expectation and maximization steps can be obtained from e.g. Mrode and Thompson [14]. Convergence of the EM algorithm was assumed when the condition

(6) $$ \sqrt{\frac{\left(\mathbf{B}_{n-1}^{}-\mathbf{B}_{n}^{}\right)^{\top} \left(\mathbf{B}_{n-1}^{}-\mathbf{B}_{n}^{}\right)}{\mathbf{B}_{n}^{\top} \mathbf{B}_{n}^{}}}<\epsilon,  $$

was fulfilled [15], where *ε*=10^−8^ and $\mathbf {B}_{n}=\left [\begin {array}{ccc}\hat {{\sigma _{1}^{2}}}&\hat {{\sigma _{2}^{2}}}&\hat {{\sigma _{e}^{2}}}\end {array}\right ]^{\top }$ is the vector of estimates of the variance components in the *n-th* iteration. False discovery rates were computed with the help of the R-package qvalue [16]. In the case of *p*-values from RLRT test statistics, the ’bootstrap’ option was used to estimate *π*_0_, as suggested by Storey [17].

## Results and Discussion

### Simulated data sets

First, the results of the RLRT for the blending error variance component are shown for the case of the validity of the null hypothesis (${\sigma _{2}^{2}}=0$). Here, a uniform distribution of *p*-values can be observed on the interval [0,0.5) as expected (see Fig. 2, topright). The Distributions of log-estimates of ${\sigma _{2}^{2}}$ (Fig. 2, left panels from top to bottom) show an increasing proportion of large values, in full accordance with the increase in the simulated proportions of transcripts with a relevant blending error variance (which was 0, 1/3 and 1). The corresponding *p*-values (right panels of Fig. 2, top to bottom) fairly mirror the same trend. The estimates for $\hat {\pi }_{1}$ approximated the simulated proportions of affected transcripts well. However, when it came to the identification of individual transcripts, their number clearly lagged behind the proportions present in the data. Corresponding results are shown in Table 2.

**Table 2:** Number of transcripts with non-zero blending error variance

Number or	Data set							
proportion of	Simulated				Experimental			
transcripts	s1	s2	s3		Mouse	Rat	Bee	Human
Total	9000	9000	9000		18646	15923	14400	43256
Crit. >8	9000	9000	9000		8554	6264	13761	12348
Sign VC	1	1794	6704		4329	6	7093	0
$\hat {\pi }_{1}$	0.005	0.295	0.918		0.75	0.29	0.68	0.40

Results of the restricted likelihood ratio tests of the hypothesis $\mathbf {H}_{0}:{\sigma _{2}^{2}}=0$ for transcripts exceeding the minimum expression level (crit. >8). Numbers of transcripts with a significant variance component for imperfect blending (sign VC) were counted according to the FDR correction level of 5 *%*. $\hat {\pi }_{1}$ is the estimated proportion of transcripts with ${\sigma _{2}^{2}}>0$. Simulated data sets s1, s2 and s3 refer to scenarios where none, one third, and all transcripts were associated with a non-zero blending error variance component

**Fig. 3:**
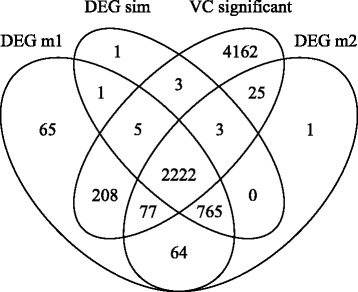
Sets of DEGs and transcripts with significant RLRT for repeatedly simulated data. Sets of differentially expressed transcripts (DEGs) for both models and coincidences of transcripts with a significant variance component for imperfect blending. These were averaged over 100 repetitions of the simulated experiment based on the mouse design and variance components ${\sigma _{t}^{2}}=0.017,{\sigma _{b}^{2}}=0.094$ and ${\sigma _{z}^{2}}=2.7$ (all transcripts with effects for imperfect blending). The average counts of the sets of differentially expressed transcripts are labeled with ’DEG m1’ for the null model, ’DEG m2’ for the full model, ’VC significant’ for transcripts with a significant blending error variance, and ’DEG sim’ for the transcripts simulated as differentially expressed

Differences in both models’ abilities to find differential expression in the simulated data sets were also observed (Table 3). The null model yielded an average of 3407 expressed transcripts declared as differentially expressed, compared to 3157 from the full model. The average shared number is 3128, but the 3000 simulated as differentially expressed in a total of 9000 transcripts was clearly outbid by both models. Figure 3 shows the average numbers of four sets of transcripts and their intersections: the set of transcripts with a simulated differential expression, one set of transcripts identified as differentially expressed for each of both models, and the set of transcripts, which were identified as connected with an attributable (larger than zero in terms of FDR) blending error variance. Upon counting the numbers in the intersection regions which corresponded to true discoveries, a similarly high power for both models was observed. Only 7 (m1) and 10 (m2) of the transcripts simulated as differentially expressed have not been found. But, adding the numbers which correspond to false discoveries yielded a value of (1+25+64+77)/6000=0.028 for m2 and (65 +208 + 64 + 77)/6000=0.069 for m1. This is clearly larger than 5 *%*, the chosen level of permitted false discoveries. The number of transcripts incorrectly labelled as differentially expressed in the group of transcripts with a significant blending error variance was inflated by a factor of about three for m1 (285) in comparison with m2 (102).

**Table 3:** Detection of differential expression by model and data set

Number of	Data set							
transcripts	Simulated				Experimental			
identified	s1	s2	s3		Mouse	Rat	Human	
m1 & m2	3112	3119	3128		3344	1636	787	
m1	48	113	279		504	141	350	
m2	4	13	29		516	12	154	

Number of transcripts identified as differentially expressed at an FDR of 5 *%* by data set and model. Simulated data sets s1, s2, and s3 refer to scenarios where none, one third, and all transcripts were associated with a non-zero blending error variance component. The number of transcripts identified with both models is indicated by m1 & m2, transcripts identified solely with the null model (m1) or the full model (m2) are shown in the second-to-last and the last line

Furthermore, in a series of simulations, the pooling technical variance ${\sigma _{z}^{2}}$ was varied within the range of (0,2.7]. A plot of the obtained estimates of ${\sigma _{2}^{2}}$ against the simulated values ${\sigma _{z}^{2}}(e^{{\sigma _{b}^{2}}}-1)$ (see Additional file 2) shows nearly perfect consistency. The exception is some upward bias for very small simulated values, which can be attributed to the well-known properties of the REML-method [18].

Therefore, it can be concluded at the very least that tests for differential expression with the m1 model tend to be too optimistic, depending on the given experimental conditions. To summarize, should the model contain the additional random effect of imperfect blending, the statistical analysis yields results which agree very well with the simulated characteristics.

### Experimental data

**Fig. 4:**
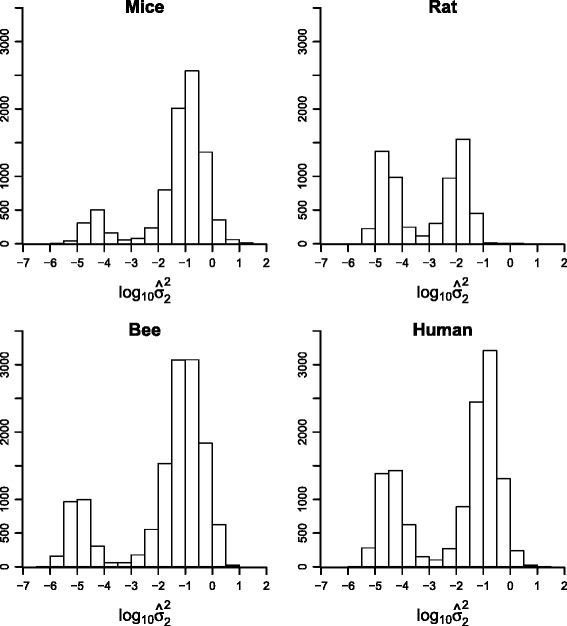
Estimates of blending error variance in empirical data. Histogram of log-estimates of the variance component ${\sigma _{2}^{2}}$ for the experimental data sets mouse, rat, bee, and human

**Fig. 5:**
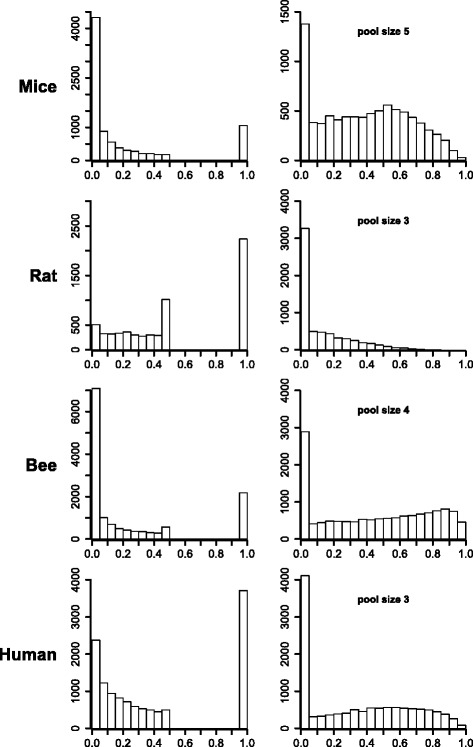
*p*-values of RLRT and variance ratios. For each experimental data set, a histogram of *p*-values of the likelihood ratio test statistic for the test of $H_{0}: {\sigma _{2}^{2}}=0$ are shown *(left)*, as well as histograms of the variance components for imperfect blending, expressed as the proportion of the total variance *(right)* of a standard observation. y-axis: count of transcripts

Histograms of log-transformed estimations of the variance components due to imperfect blending are shown in Fig. 4. Estimates range from nearly zero (10^−6^) to less than one hundred (10^2^). A clear bimodal distribution can be observed in all cases, where the left part of each distribution (values less than approximately 10^−3^) represents very small values close to zero while the other part represents more substantial values. In the mouse and the bee data, the proportion of transcripts with substantially large values clearly exceeds the proportion of small values. For the human data, the proportion of small estimates also prevails somewhat, while a balance between minor and substantial values can be observed for the rat data. This is also reflected in the average (over all transcripts) of all three variance components obtained with the reduced (m1) and the full (m2) models, as shown in Table 4. In light of the averages, the inclusion of a blending error variance had the consequence of a more or less reduced residual variance, most pronounced in the mouse and honey bee data. In the human data, the average residual variance remained almost constant, yet the average biological variance decreased - a phenomenon not observed in the other data sets. Distributions of the size of ${\sigma _{2}^{2}}$ relative to the total variance of a standard observation - with respective pool sizes of 5, 3, 4, and 3 for mouse, rat, bee and human data are given in Fig. 5 (right, top to bottom). All distributions exhibit a clear spike near zero, followed by estimates that nearly exceed the full range of variance ratios. The rat data are an exception; hardly any values larger than 0.6 were observed.

**Table 4:** Mean estimated variance components

Mean estimated	Experimental data set/model used										
variance	Mouse			Rat			Bee			Human	
component	m1	m2		m1	m2		m1	m2		m1	m2
${\sigma _{e}^{2}}$	0.037	0.017		0.010	0.009		0.104	0.035		0.062	0.060
${\sigma _{1}^{2}}$	0.109	0.109		0.024	0.024		0.031	0.033		0.105	0.055
${\sigma _{2}^{2}}$	-	0.295		-	0.011		-	0.215		-	0.155

Estimated variance components for residuals (${\sigma _{e}^{2}}$), biological effects (${\sigma _{1}^{2}}$), and imperfect blending (${\sigma _{2}^{2}}$) - averaged over all analyzed transcripts for the null model (m1) and the full model (m2)

These impressions are mirrored by the distributions of *p*-values from RLRT-tests for the hypothesis of a non-existing (${\sigma _{2}^{2}}=0$) blending error variance (left panels in Fig. 5, top to bottom). The number of individual transcripts, which could be associated with a non-zero blending error variance at a false discovery rate of 5 *%*, varied strongly between data sets. There were 4329 of such transcripts in the mouse data and 7093 in the honey bee data, while only six were identified in the rat data and none at all in the human data (Table 2). These high numbers are consistent with considerable estimates for the fraction ($\hat {\pi }_{1}$) of non-zero variances in mouse ($\hat {\pi }_{1}=0.75$) and honey bee ($\hat {\pi }_{1}=0.68$) data (Table 2). Note that the respective estimated proportions were $\hat {\pi }_{1}=0.29$ and $\hat {\pi }_{1}=0.40$ in the rat and human data (Table 2), also indicating the existence of non-zero blending error variances in these two data sets, though almost no particular non-zero variance could have successfully been identified at the chosen false discovery rate of 5 *%*.

Counts of differentially expressed transcripts detected with both models are shown in Table 3. About half of all transcripts analyzed were declared differentially expressed in the mouse data. About five hundred were exclusively detected with one of both models: 504 with the null model and 516 with the full model. The list of the top 100 transcripts - ranked by their *p*-values - showed a large dissimilarity as indicated by a value of 0.11 for Kendall’s correlation test. In the rat data, 1636 differentially expressed transcripts were jointly identified by both models, while 141 were solely found with the help of m1 and 12 with m2. No numbers appear in Table 3 for the honeybee data, as no differentially expressed transcripts were found. Finally, there were 1137 differentially expressed transcripts from the null model in the human data, from which only 787 were ’confirmed’ by the full model.

## Conclusions

In light of the large numbers of blending error variances diagnosed as greater than zero in the mouse and honey bee data, the practical relevance of this second kind of technical error has been clearly demonstrated. In both other data sets, estimates of $\hat {\pi }_{1}$, the proportion of positive blending error variances, may be taken as an indicator of their existence, though hardly any particular values could be identified, presumably due to a lack of power. As demonstrated mainly by simulation, there are also consequences for the detection of differentially expressed transcripts, in which the nominal FDR-level was shown to be too optimistic when the blending error variance was not taken into account. Therefore, we strongly recommend the application of adequate models (as described in [3]) including random blending effects and their variances when observations from pools of different sizes are to be jointly analysed.

## Availability of supporting data

The mouse and honey bee data sets have been deposited at the Gene Expression Omnibus (GEO) website (www.ncbi.nlm.nih.gov/geo), under the joint accession no. GSE72944. The human data is part of the data set GSE6112 and the rat data has the GEO accession no. GSE2331.

## Additional files

Additional file 1
**Matrices for EM-REML and Mixed model equations.** This file shows various matrices for the experimental data sets in detail. These matrices are explained in the Materials and Methods section. (PDF 48 kb)

Additional file 2
**Comparison of simulated and estimated blending error variance.** Plot of the average estimated variance components ${\hat \sigma _{2}^{2}}$ versus simulated values ${\sigma _{z}^{2}}(e^{{\sigma _{b}^{2}}}-1)$. In various simulation runs, the pooling technical variance ${\sigma _{z}^{2}}$ was altered in the range of (0,2.7] to evaluate whether the approximation in Eq. (3) is applicable for our purposes. Numbers of individuals in a pool were randomly chosen. For each number, as many individuals were artificially blended into a pool and an equally sized pool of controls was opposed. Estimates and simulated values agree very well; some bias for small values can be attributed to the EM-REML algorithm used for variance component estimation. (PDF 9 kb)

## Abbreviations

EM: Expectation maximization
FDR: False discovery rate
REML: Restricted maximum likelihood
RLRT: Residual likelihood ratio test
RNA: Ribonucleic acid
