# Supplementary figures and images for: On the relevance of technical variation due to building pools in microarray experiments
Source: BMC Genomics. 2015 Dec 1;16:1027. doi: 10.1186/s12864-015-2055-6 (PMC4667463; doi:10.1186/s12864-015-2055-6)

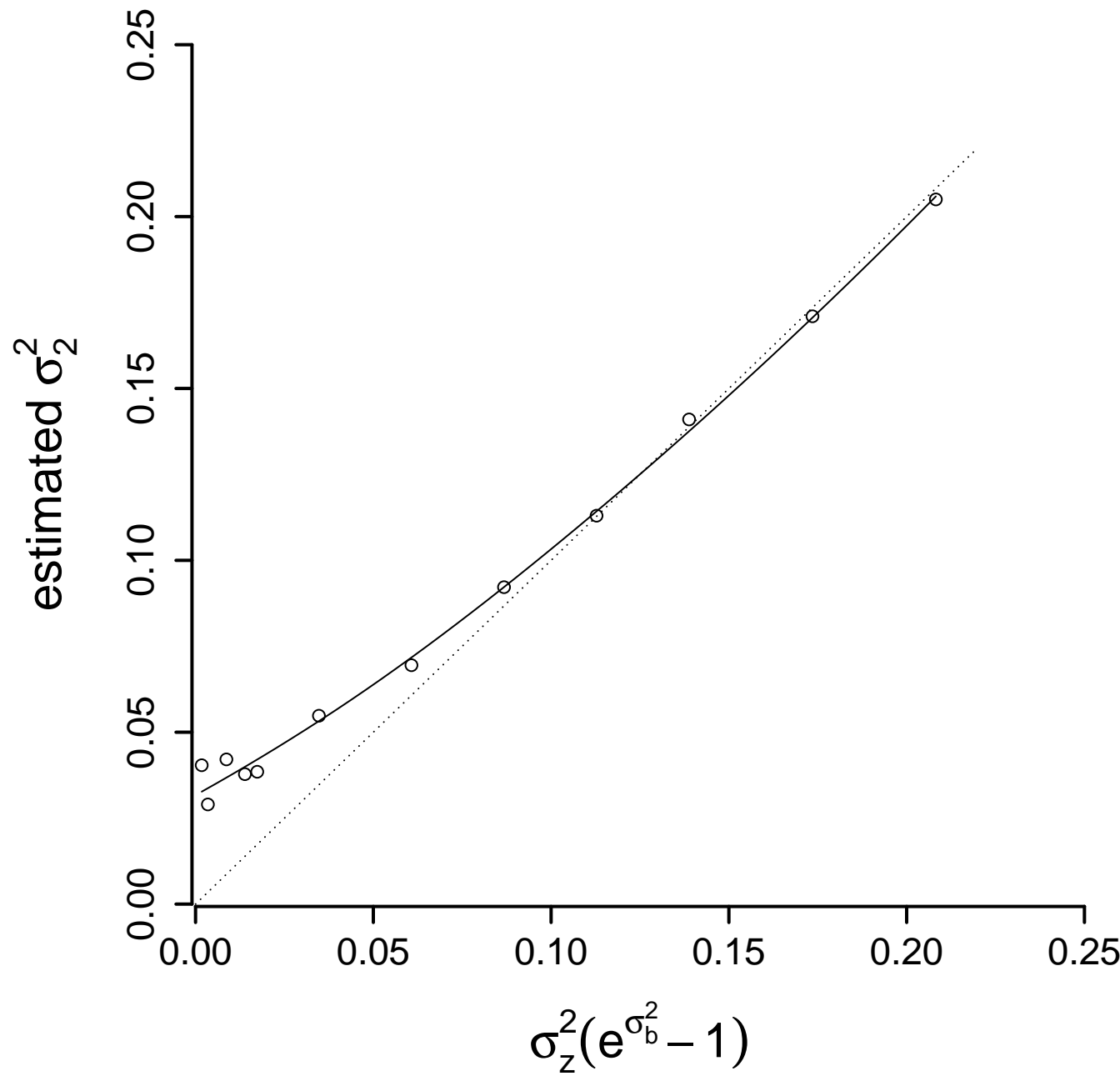

Supplement: Additional file 2 — Comparison of simulated and estimated blending error variance. Plot of the average estimated variance components ${\hat \sigma _{2}^{2}}$σ^22 versus simulated values ${\sigma _{z}^{2}}(e^{{\sigma _{b}^{2}}}-1)$σz2(eσb2−1). In various simulation runs, the pooling technical variance ${\sigma _{z}^{2}}$σz2 was altered in the range of (0,2.7] to evaluate whether the approximation in Eq. (3) is applicable for our purposes. Numbers of individuals in a pool were randomly chosen. For each number, as many individuals were artificially blended into a pool and an equally sized pool of controls was opposed. Estimates and simulated values agree very well; some bias for small values can be attributed to the EM-REML algorithm used for variance component estimation. (PDF 9 kb) [file 12864_2015_2055_MOESM2_ESM.pdf]
